# PK/PD modeling of Ceftiofur Sodium against *Haemophilus parasuis* infection in pigs

**DOI:** 10.1186/s12917-019-2008-4

**Published:** 2019-08-01

**Authors:** Xiao-dong Li, Sheng-Qing Chi, Li-Yun Wu, Can Liu, Tong Sun, Juan Hong, Xun Chen, Xiao-Gang Chen, Guan-Song Wang, Dao-Jin Yu

**Affiliations:** Fujian Key Laboratory of Traditional Chinese Veterinary Medicine and Animal Health, Fujian Agriculture and Forestry University, Fuzhou, China

**Keywords:** Ceftiofur sodium, *Haemophilus parasuis*, PK/PD model, Ex vivo bactericidal activity, Glässers disease

## Abstract

**Background:**

Ceftiofur Sodium is widely used in China. Our aim was to determine Ceftiofur Sodium activity and optimize dosing regimens against the pathogen *Haemophilus parasuis* using an in vitro and ex vivo pharmacokinetics/pharmacodynamics modeling approach. By adopting these strategies, we wanted to extend the effective life of Ceftiofur Sodium in reduce drug-resistance in pigs.

**Results:**

We established an *H. parasuis* infection model in pigs, and assessed the pharmacokinetics of Ceftiofur Sodium in both healthy and infected animals. After Ceftiofur Sodium (10 mg/kg, i.m.) administration to healthy and *H. parasuis*-infected pigs, plasma based desfuroylceftiofur (a metabolite of Ceftiofur Sodium) was measured by High Performance Liquid Chromatography. The pharmacokinetics of Ceftiofur Sodium (desfuroylceftiofur) was consistent with a two-compartment open model, with first-order absorption. We observed no significant differences (*P* > 0.05) in pharmacokinetic parameters between healthy and infected pigs. Pharmacodynamics data showed that Ceftiofur Sodium was highly inhibitory against *H. parasuis*, with MIC, MBC, and MPC values of 0.003125, 0.0125 and 0.032 μg/mL, respectively. Desfuroylceftiofur in plasma also had strong bactericidal activity. Almost all *H. parasuis* cultured in plasma medium of Ceftiofur Sodium-inoculated healthy pigs, at each time point, were killed within 24 h. A weaker antibacterial activity was measured in infected-pig plasma medium at 18, 24, 36, and 48 h, after Ceftiofur Sodium inoculation. Pharmacokinetic parameters were combined with ex vivo pharmacodynamic parameters, and the bacteriostatic effect (36.006 h), bactericidal effect (71.637 h) and clearance (90.619 h) within 24 h, were determined using the Hill equation. Dose-calculation equations revealed the optimal dose of Ceftiofur Sodium to be 0.599–1.507 mg/kg.

**Conclusions:**

There were no significant differences in Ceftiofur Sodium pharmacokinetic parameters between healthy and infected pigs, although pharmacokinetics/pharmacodynamics fitting curves showed obviously differences. The optimal dose of Ceftiofur Sodium was lower than recommended (3 mg/kg), which may provide improved treatments for Glässers disease, with lower drug-resistance possibility.

## Background

Globally, Glässers disease (GD) is a fatal swine disease, and is one of the main causes of nursery pig deaths on large-scale farms [1–3]. The disease is characterized by fibrinous polyserositis, polyarthritis, meningitis and acute pneumonia (without polyserositis) [4, 5], and is associated with high morbidity and mortality [1, 6]. GD is caused by a gram-negative bacillus *Haemophilus parasuis* [1] which is an opportunistic pathogen residing in the nasal and oral cavities of most pigs. The *bacillus* is primarily benign [7, 8], however it causes disease when pigs become stressed [1].

Antimicrobials have been used to treat GD, however this causes drug resistance due to the widespread, non-standard use of drugs on intensive pig breeding farms [9]. Additionally, several *H. parasuis* genes, resistant to multiple antimicrobials, have been identified in this pathogen [10–17]. When high development costs, safety risks and rapid pathogen resistance to new drugs are all considered, it is advantageous to exploit existing antibacterial drugs by designing optimal treatments to avoid pathogen exposure to sub-lethal drug concentrations [18, 19].

Ceftiofur Sodium (CS) is an animal-specific, third-generation cephalosporin, widely used in China and abroad for its activity against most Gram-negative and some Gram-positive bacteria [20–22]. It is rapidly metabolized to desfuroylceftiofur (DFC) upon intravenous injection. It has been shown that CS and DFC have similar activities against Gram-negative bacteria, with minimum inhibitory concentrations (MIC) differing by less than one dilution factor [23]. Studies have been conducted on the pharmacodynamics (PD) of CS to some pathogens like *Klebsiella pneumonia* and *Staphylococcus aureus* [24]. Similarly, the pharmacokinetics (PK) of CS has also been reported in swine and calves [25–27]. PK and PD data has also been generated for CS activities in goat, against *Mannheimia haemolytica* and *Pasteurella multocida* [28, 29], and in cattle against *Escherichia coli* [30]. However, PK/PD modeling of CS against *H. parasuis* has not yet been studied.

According to the relationship between PK and PD, a reasonable dosing drug regimen can be established to maximize clinical efficacy and to minimize the selection pressures of bacterial resistance and the spread of drug-resistant pathogens [31–33]. The ex vivo model uses plasma or tissue percolates collected over a time series after drug administration (oral, intravenous or intramuscular injection) instead drug solution of directly in vitro PD measurement. Combined with in vivo PK measurements, a corresponding dosing regimen can be more rationally formulated.

In this study, PK/PD parameters of CS activity against *H. parasuis* were explored through the ex vivo PD parameters of CS and in vivo PK parameters. Differences in PK parameters between healthy and infected pigs were also compared, and a corresponding dosing regimen proposed, which we wish to help exert best therapeutic effect, reduce prevalence of bacterial resistance, and prolong the lifespan of CS in the clinic.

## Results

### Growth curve of *H. parasuis* in TSB medium

**Fig. 1:**
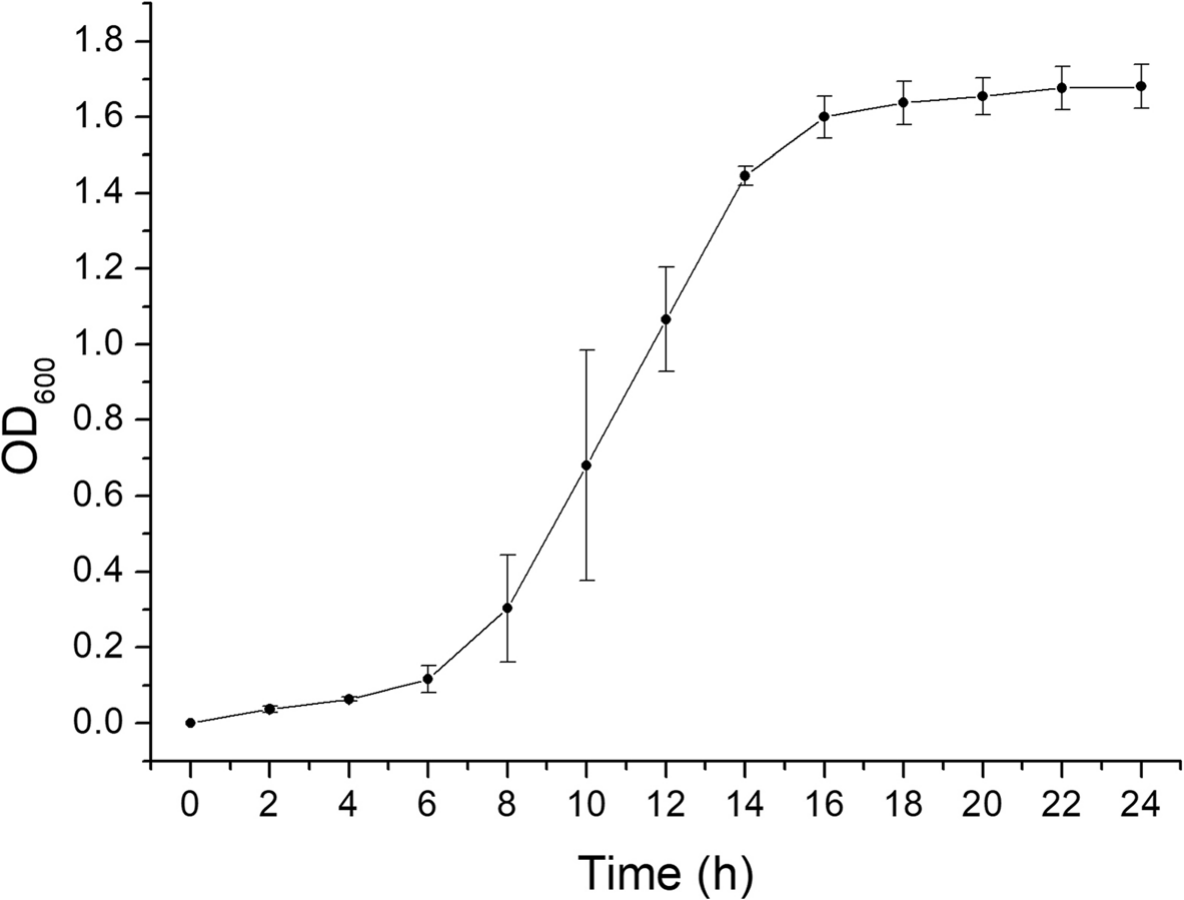
The growth curve of *Haemophilus parasuis* in TSB medium. Density of bacteria was represented by absorbance value under 600 nm (OD_600_). Square dots indicate the mean of triplicate measurements of densities and the bars indicate standard deviation (% SD)

As shown in Fig. 1, *H. parasuis* growth in TSB medium conformed to the logistic growth model. The exponential period was at approximately 6–16 h, and the T_1/2K_ was at approximately 12 h.

### Symptoms of *H. parasuis* infection

After a 2–4 h inoculation, piglets experienced increased body temperatures, vomiting, runny noses, depression, reduced appetites, difficulty in breathing, redness of skin, messy coat hair, muscle tremors, instability on their legs (followed by lameness), slight cyanosis in the nose, ear tip and limbs ending, orbital edema and ataxia. These symptoms indicated that the *H. parasuis* infection model had been established.

### The PK of CS in healthy and infected piglets

Specificity tests showed that the DFC peak was at approximately 13.5 min, with no interference impurity peak nearby. This method also had a high precision (intra-day RSD < 4.10% and inter-day RSD < 8.92%) and DFC recovery (91.07–94.48%). A standard curve was established; y = 0.7183x - 0.05117 (where y: Response (mA·min), x: DFC concentration (μg/mL)) with R^2^ = 0.9995.

**Fig. 2:**
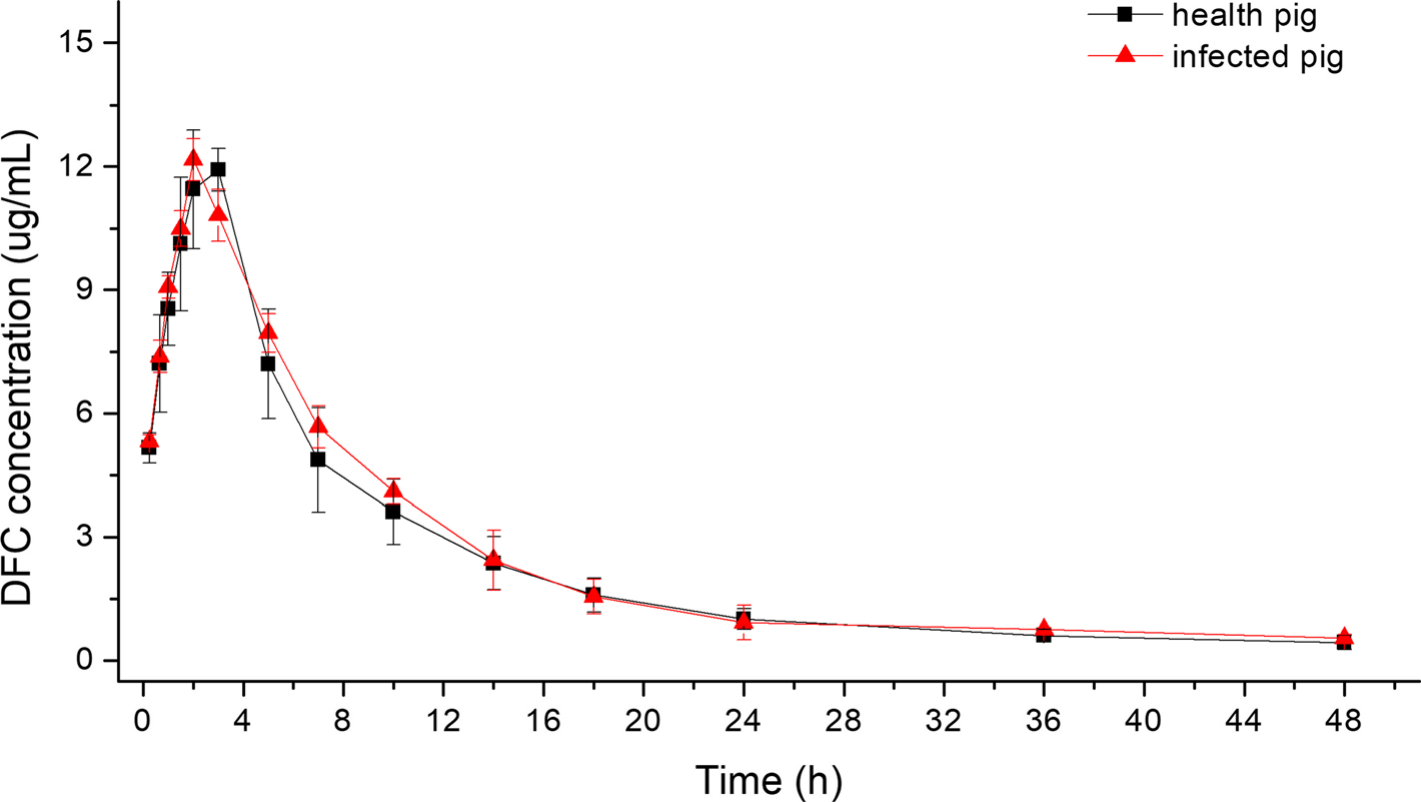
The concentration-time curve of DFC in both health and infected pigs after intramuscular injection of CS. Dots indicate the mean of concentration in health (black square) and infected pigs (red triangle), and the bars indicate standard deviation (% SD)

As shown in Fig. 2, DFC concentrations were measured at different times, in both healthy and infected pigs. DFC concentrations changed similarly between healthy and infected groups, and there were no significant differences (*P* > 0.05) between healthy and infected groups, at each time point. However, DFC concentrations in infected pigs increased a little faster than in healthy pigs.

Analyses by WinNonlin, using the Atrioventricular Model, showed that the PK of CS (DFC) after intramuscular injection was consistent with a two-compartment open model, with first-order absorption. The main PK parameters are shown in Table 1, after curve fitting. These parameters showed the same patterns as with measurement curves. There were no significant differences between the PK parameters of the infected group and the healthy group (P > 0.05), suggesting that the PK of CS in these groups were similar.

**Table 1:** Pharmacokinetic parameters of CS (10 mg/kg, i.m.) after fitting two-compartment open model with first-order absorption

Parameter	Unit	Healthy pigs (*n* = 8)	Diseased pigs (*n* = 6)
Ka	1/h	0.489 ± 0.061^a^	0.537 ± 0.054^a^
AUC	h·μg/mL	125.646 ± 9.895^a^	126.498 ± 13.752^a^
AUMC	h^2^·μg/mL	2201.958 ± 829.817^a^	1703.975 ± 442.930^a^
MRT	h	15.503 ± 7.001^a^	11.436 ± 2.547^a^
C_max_	μg/mL	11.236 ± 0.603^a^	11.504 ± 0.575^a^
T_max_	h	2.358 ± 0.286^a^	2.226 ± 0.146^a^
T_1/2Ka_	h	1.456 ± 0.172^a^	1.281 ± 0.143^a^
T_1/2Ke_	h	17.550 ± 8.830^a^	11.247 ± 2.930^a^
V_d_/F	L/kg	2.402 ± 0.999^a^	1.649 ± 0.482^a^
CL/F	mL/h/kg	80.010 ± 6.115^a^	79.837 ± 8.640^a^

Ka, absorption rate constant; AUC, area under the concentration-time curve after every administration; AUMC, the area under the first moment of the plasma concentration-time curve; MRT, presented mean residence time; C_max_, the maximum concentration during the dosage interval; T_max_, the time to reach peak concentration; T_1/2Ka_, absorption half-life; T_1/2Ke_, elimination half-life; V_d_/F, volume of distribution; CL/F, systemic clearance. Different lowercase letters indicate a significant difference (*P* < 0.05)

### The PD of CS in *H. parasuis*

CS strongly inhibited *H. parasuis*, with a CS MIC of 0.03125 μg/mL in TSB medium. The MBC and MPC were 0.0125 and 0.0320 μg/mL, respectively. We also obtained results from plasma medium; MIC and MBC in plasma medium were 0.00625 and 0.0250 μg/mL, respectively.

**Fig. 3:**
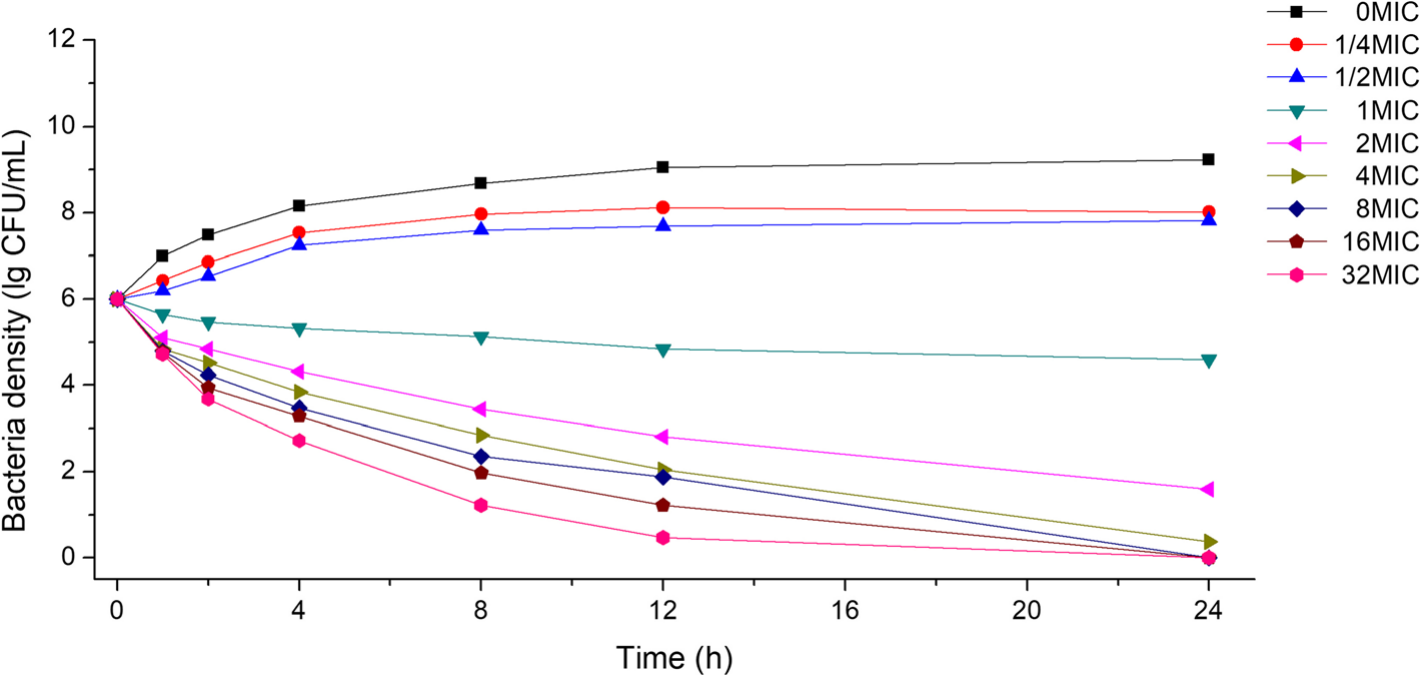
The 24 h inhibition curve of CS against *Haemophilus parasuis*. Density of bacteria was represented by logarithm of actual concentration (CFU/mL)

As shown in Fig. 3, bacterial densities decreased rapidly after CS addition, at a density above (and including) the MIC. Similarly, bacterial densities decreased for a long time with dropping slopes. These observations indicated that CS had long term inhibitory effect against *Parasuis*, but these effects gradually diminished over time, suggesting CS inhibition of *Parasuis* was in part dose-related.

The PAE of CS in *H. parasuis* increased gradually with increasing CS concentrations, but the degree of enhancement was weak. PAE were 1.06, 1.12, and 1.46 in 1, 2, and 4 MIC, respectively.

**Fig. 4:**
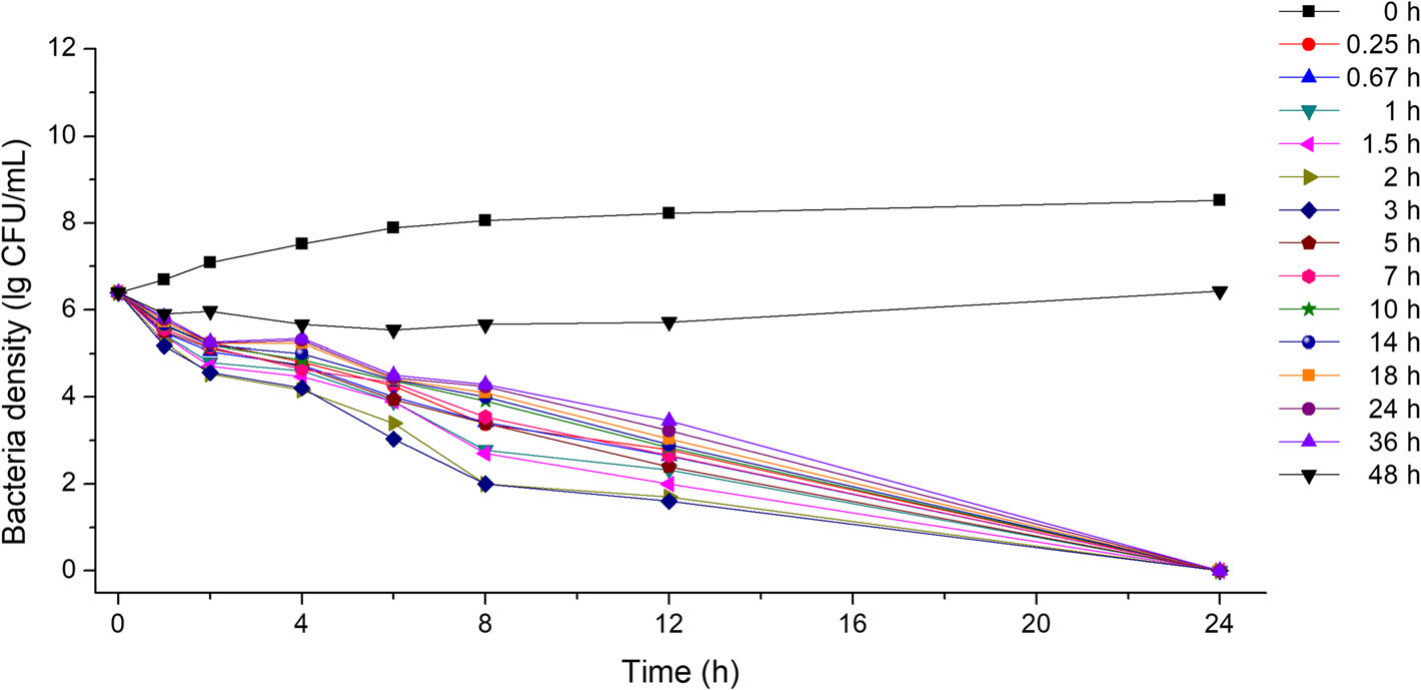
Ex vivo bactericidal curves of DFC in the plasma of healthy pigs against *Haemophilus parasuis.* Density of bacteria was represented by logarithm of actual concentration (CFU/mL)

**Fig. 5:**
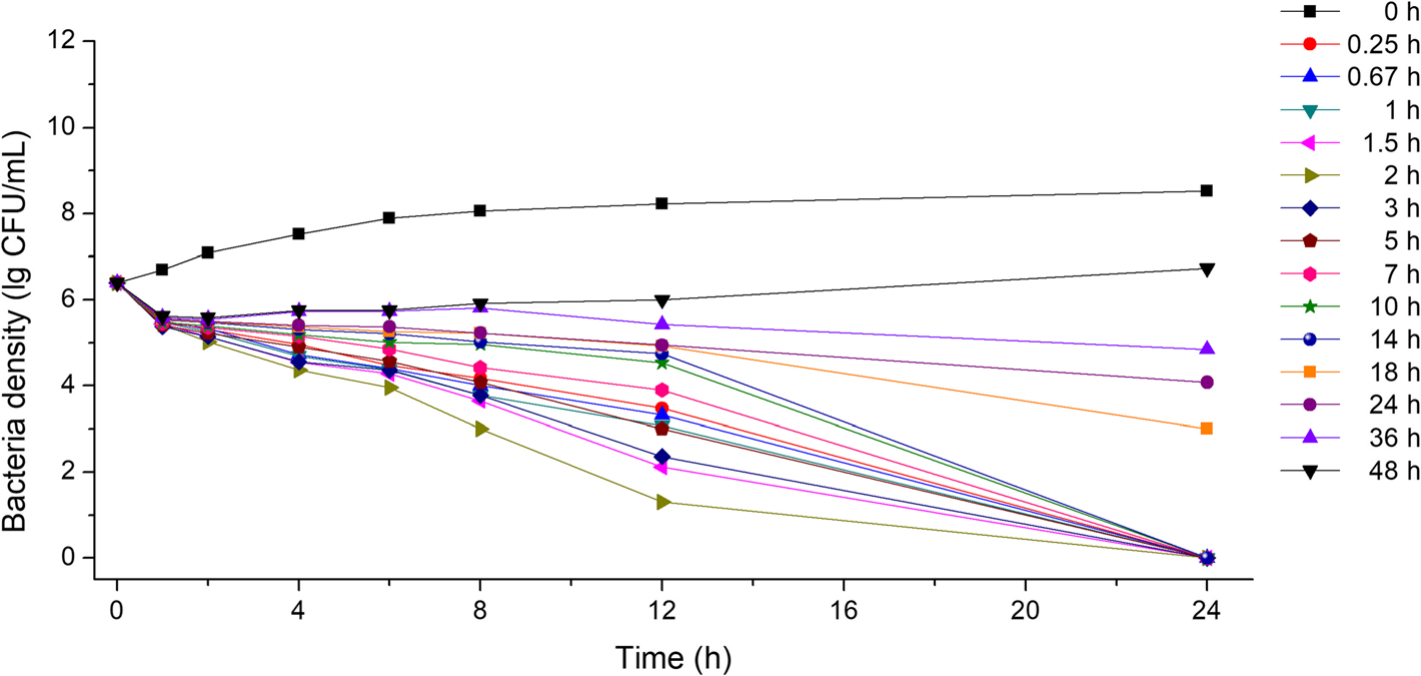
Ex vivo bactericidal profiles of DFC in the plasma of diseased pigs against *Haemophilus parasuis.* Density of bacteria was represented by logarithm of actual concentration (CFU/mL)

In vitro bactericidal curves, at different time points, are shown in Figs. 4 and 5. DFC had strong bactericidal activity in the plasma of healthy pigs (Fig. 4). Almost all bacteria were killed after 24 h of culture, with the 48-h sample exhibiting weak antibacterial activity. Plasma samples from infected pigs had weaker antibacterial activity at 18, 24, 36, and 48 h (Fig. 5). This indicated that the DFC activity was different between healthy and infected pigs, although PK data were similar between the groups.

### The establishment of a PK/PD model and dosing regimen

**Fig. 6:**
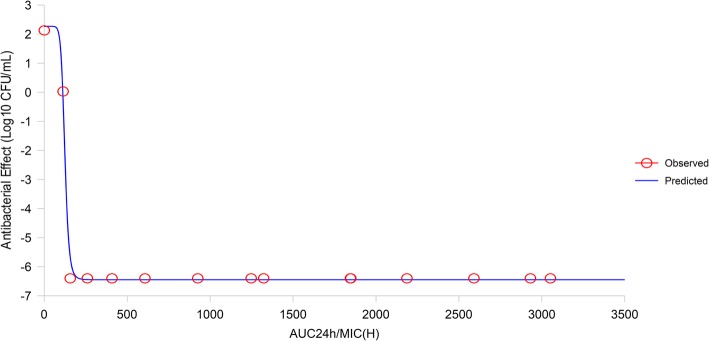
Curve-fitting results for healthy pigs

**Fig. 7:**
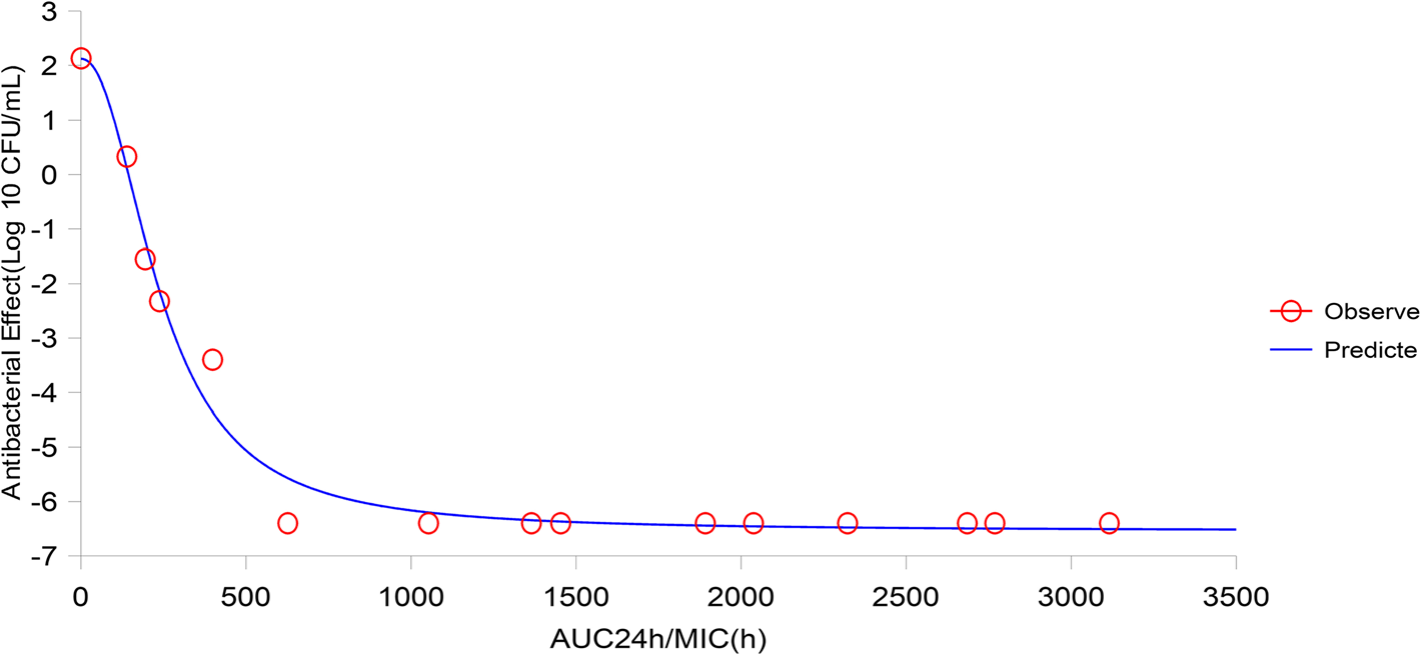
Curve-fitting results for diseased pigs

The PK/PD parameters describing the bactericidal effects of CS against *H. parasuis* are reflected by AUC/MIC values. Specifically, when plasma was inoculated with bacteria at different time points, the mean ex vivo value of AUC/MIC was the product of the culture time and diluted concentration as the indirect in vivo AUC divided by the MIC measured in vitro. WinNonlin was used to fit ex vivo PD data and PK/PD parameters into the Sigmoid E_max_ model (the Hill equation) (Figs. 6 and 7) to show the relationship between ex vivo AUC_24 h_/MIC and antibacterial efficacy (Table 2). The effects of CS were established in healthy pigs more quickly than the infected group (Figs. 6 and 7). CS exhibited a similar bacterial inhibition at different degrees, with bacteriostatic action at 28.406 h, bactericidal action at 29.516 h, and bacteriophagous action at 29.913 h. However, bacterial inhibition was different in infected pigs, with bacteriostatic action at 36.006 h, bactericidal action at 71.637 h, and bacteriophagous action at 90.619 h (Table 2). When the AUC_24 h_/MIC reached 90.619 h, *H. parasuis* was eliminated (the parameter values of CS to achieve antibacterial effects refer to the PD data of healthy pigs, and the parameter values that achieved sterilization effects refer to the PD data of infected pigs). By fitting the Hill equation to infected pig data, dose-calculation equations revealed the optimal dose of CS to be 0.599–1.507 mg/kg.

**Table 2:** Results for fitting of the Hill equation

Parameter	Healthy pigs	Infected pigs
E_max_ (lg CFU)	−6.449	− 6.543
EC_50_ (h)	124.247	241.774
E_0_ (lg CFU)	2.268	2.120
N	10.000	2.171
(C_e E = 0_) AUC_24 h_/MIC for bacteriostatic action (h)	28.406	36.006
(C_e E = -3_) AUC_24 h_/MIC for bactericidal action (h)	29.516	71.637
(C_e E = -4_) AUC_24 h_/MIC for bacteriophagous action (h)	29.913	90.619
Dose (C_e E = 0_) (mg/kg)	0.473	0.599
Dose (C_e E = -3_) (mg/kg)	0.492	1.191
Dose (C_e E = -4_) (mg/kg)	0.499	1.507

AUC represents the area under the curve; MIC is the minimum inhibitory concentration; E_0_ represents the maximum difference in the logarithm of the bacterial count after 24 h of bacterial culture in the blank group; E_max_ is the maximum difference in the logarithm of the bacterial count after 24 h of bacterial culture in the serum sample; EC_50_ represents the AUC/MIC when reaching 50% of the maximum antibacterial effect in the serum sample; N is the slope of the equation (no unit)

## Discussion

The determination of dosing regimens for sick piglets is primarily based on PK data of reference drugs in healthy pigs. However, the absorption, distribution and metabolism of drugs are different between healthy and diseased animals, and so too is the response of animals to these drugs. These differences can cause changes in PK characteristics of drugs in animals, however traditional formulation methods do not consider these changes in diseased animal physiology [34]. Drug regimens will become more accurate and effective if PK differences between healthy and diseased animals are determined.

*H. parasuis* mainly infects pigs aged between 2 and 16 weeks (especially piglets aged 5–8 weeks). In the present study, 6-week-old nursery pigs were selected to test the infection characteristics of *H. parasuis*. The inoculation dose in our model was 3.0 × 10^9^ CFU/mL, with pig mortality reaching 50% after 4 h, similar to Wang et al. [35]. In their study, mortality reached 40% upon intraperitoneal inoculation with 3.4 × 10^9^ CFU/mL of the *H. parasuis* S4 strain. At 2–4 h post inoculation, the symptoms of vaccinated pigs were similar to those of pigs infected with *H. parasuis*, as noted by Yin et al. [36] and Oliveira et al. [37]. Plasma from both healthy and infected pigs was the main (convenient and fast) reagent for ex vivo testing [29, 38, 39], however, other materials, like ileum content [39], were also previous used, which depends on the action site of the pathogen.

We investigated the PK characteristics of CS (10 mg/kg, i.m.) in pigs infected with *H. parasuis,* and also healthy pigs. The PK of healthy and infected pigs corresponded to a two-compartment open model, with first-order absorption. The peak CS concentration reached 11.236 μg/mL at 2.358 h in healthy pigs, the absorption half-life was 1.517 h, the elimination half-life was 17.550 h, and the AUC was 125.646 h·μg/mL. These data are different to Hu et al. [40], and may due to the pig’s age, the dose administered (10 mg/kg in this study but 5 mg/kg in Hu et al. [40]) or different analysis software.

PK differences between healthy and infected pigs were not significant (*P* > 0.05), suggesting that the time, speed, degree, bioavailability and distribution of CS in healthy and infected pigs were not significantly different. Similarly, Zhang et al. [41] analyzed the PK of tilmicosin in both healthy and *H. parasuis* infected pigs, and found no significant differences between the PK profiles in the two groups. However, Gao et al. [42] observed leukopenia and hypoglycemia in *H. parasuis*-infected pigs, with varying degrees of injury to liver and kidney. These conditions change the distribution, absorption and elimination of the drug in a pigs’ body [42]. Our study showed some differences to Gao et al. [42], possibly because: (i) PK observations were performed a short time after artificial infection with *H. parasuis* and (ii) in our study *H. parasuis* did not cause similar kidney and liver damage.

For a time-dependent drug, the time that CS concentrations were above the MIC (T > MIC) should be well determined in its PK/PD modeling. However, plasma concentrations in the in vitro tests were constant, such that T > MIC is hardly to be accurate. According to the CS curve against *H. parasuis*, we found an obvious time depending inhibition activity same with a partly concentration-dependent pattern (Fig. 3), for which the AUC_24 h_/MIC as a suitable PK/PD parameter for CS [43]. For drugs like CS and other β-lactam antimicrobials, the key to their antibacterial effectiveness is their effective duration (i.e., T > MIC). Combined with mutant selection window theory (MSW theory; drug resistance is more likely to occur between MSWs), it is conducive to reducing drug resistance when we increase its concentration initially and keep it above MPC for a certain time. In the present study, bacteriostatic, 99.9% killing and 99.99% killing (completely sterilized without leading resistance) concentration of CS is 0.599, 1.191, 1.507 mg/kg and the recommended dosing regimen for CS was 0.599 mg/kg for prophylactic injection in healthy pigs and 1.507 mg/kg for pigs with symptoms. This regimen was different to the observations of Li et al. [25] and Chen et al. [12] who recorded a 5 mg/kg CS for a single dose, and also less than 3 mg/kg (suggested in prescription), which had sufficient inhibitory effects against *Streptococcus, Pasteurella* and *Actinobacillus* species. The reason for this may have been differences in age and body weight of pigs, and in the formulation of the drug-delivery plan. Equally, a standard *H. parasuis* strain was selected as the test strain; the CS MIC of this strain was extremely low, suggesting that the recommended dosing regimen may not be suitable for highly resistant strains.

In summary, our study provides a basic rational dosing regimen, but our PK/PD model mainly targets the antibacterial effects of CS. Antibacterial effects also include immune functions and defense capabilities [38]. The AUC of our PK/PD model was the product of drug concentrations and cultures at different time points. CS degradation in plasma was not considered. The *H. parasuis* strain used in our study was a standard strain, not a clinical-isolate strain. Therefore, CS was more sensitive to the MIC of *H. parasuis*, and the PK/PD parameters may have varied due to *H. parasuis* in the body. Therefore, our results should be used only for guidance in clinical dosing.

## Conclusions

This study suggests that CS strongly inhibits *H. parasuis* with low MIC, both serum and TSB broth, and PK of CS was consistent with a two-compartment open model with first-order absorption. There were no significant differences in PK parameters between healthy and infected pigs, however the PK/PD fitting curve showed obvious differences. The optimal CS dose was calculated at 0.599–1.507 mg/kg per day, which may be used as a guidance in clinical practice.

## Methods

### Test strains, animals and drugs

The standard strain of *H. parasuis* (SW124, serotype 4, isolated from a pig farm in Jianwei, Sichuan Province, China [44]) and *Enterococcus faecalis* (ATCC29212, quality control) were purchased from the China Institute of Veterinary Drug Control (Beijing, China). Bacterial strains were stored − 80 °C in tryptone soy broth medium (TSB medium) supplemented with 25% glycerol. They were recovered in TSB medium at 37 °C. Modified TSB medium and tryptone soy agar medium (TSA medium) supplemented with 5% bovine plasma and 10 μg/mL (final concentration) NAD^+^ were used in all tests. Strain cell density was measured by two approaches; Mcfarland standards and absorbance at OD_600nm_, where an optical density of 0.08–0.10 equated to approximately 1.0 × 10^8^ CFU/mL.

Twenty healthy Duroc × Landrace × Yorkshire three-way crossbred piglets were used for tests. All piglets (provided by Youlike Agriculture and Animal Development Co., Ltd. in Fujian Province, China) were of similar age (six weeks old) and similar physical status (mean body weight 14.96 ± 0.47 kg) and were antimicrobial free. Piglets were divided randomly (piglets were blindly numbered and randomly divided by Excel) into a control (*n* = 8) group and test (*n* = 6 × 2) groups [45]. Piglets were housed on a farm and fed a normal diet and water, free of antimicrobials. They were observed for one week before experimental procedures. Euthanasia procedures were performed by the administration of pentobarbital sodium (intravenously) at study end. The test farm was free of *H. parasuis* infection. The study was approved by the Research Ethics Committee of College of Animal Science, Fujian Agriculture and Forestry University (No. PZCASFAFU2018001).

A High-performance liquid chromatography (HPLC) CS standard (batch number 104666, with a purity over 98%) and a DFC (1-LLH-29-2) standard were obtained from Dr. Ehrenstorfer GmbH (Augsburg, Germany), while the TRC standard came from elsewhere (Lowell, MA, USA). CS powder for injection (batch number DVK170504) was purchased from Hebei Yuanzheng Pharmaceuticals (Hebei, China). The methods described herein were modified from Huang et al. [45]

### Growth curves of *H. parasuis* in TSB medium

100 μL *H. parasuis* growth medium (OD_600_ = 0.08–0.10) was transferred to 100 mL TSB medium and cultured at 37 °C, at 190 rpm. Every two hours, approximately 1 mL culture medium was collected and the OD_600nm_ measured to generate a growth curve of *H. parasuis* in TSB medium, by which harvested time was determined.

### The PK of CS in healthy and infected piglets

#### Piglet infection, drug administration and sampling

Exponentially growing bacteria were diluted to a McFarland turbidity of 10 (~ 3.0 × 10^9^ CFU/mL) in TSB, with 1 mL injected (i.p.) into each piglet. After injection, rectal temperatures and other clinical observations were monitored, e.g., vomiting, lameness, appetite loss, respiration rates and mental state.

10 mg/kg body weight of CS was injected (i.m.) into all piglets at 4 h after bacteria injection. Approximately 3 mL of blood was collected from the anterior vena cava before administration (0 h time-point) and later at 15 min, 40 min and 1, 1.5, 2, 3, 5, 7, 10, 14, 18, 24, 36, and 48 h post CS injections. Blood was immediately transferred to heparin tubes containing anticoagulant and mixed. Bloods were centrifuged at 2000 *g* 4 °C for 5 min and the resulting plasma supernatant was stored at − 20 °C.

#### CS plasma concentrations

DFC concentrations were measured by HPLC using the method from Li et al. [46] to represent CS plasma concentrations, as CS is rapidly metabolized to DFC in test animals.

Extraction: 0.5 mL plasma was mixed with 7 mL of 0.4% DTE-borate buffer. The mixture was incubated for 15 min at 50 °C in a water bath, with a 10 s vortexing every 3 min. Samples were then centrifuged after cooling to 25 °C, and the supernatant was collected.

Solid phase extraction: An Agilent C18 column (100 mg/3 cc) was activated and equilibrated consecutively with 3 mL methanol and ultrapure water. Extracted materials were added to the C18 column and a flow rate set at 1 mL/min. The column was then eluted with 6 mL methanol, after which the eluate was concentrated by nitrogen-blow at 45 °C. The concentrated solution was vortexed with 0.5 mL 0.01 mol aqueous ammonium solution, sonicated for 5 min, filtered through a 0.22 μm filter and prepared to test. The DFC standard was added to 0.5 mL plasma (to achieve final concentrations of 0.025, 0.05, 0.1, 0.5, 1.0, 2.5, 5.0, 10.0, and 20.0 μg/mL) and prepared with same process as samples from test groups.

HPLC: Samples were measured at 266 nm on an Agilent ZORBAX Eclipse XDB-C18 (4.6 × 150 mm, 5 μm), using a mobile phase of acetonitrile and 0.1% trifluoroacetic acid, at 1 mL/min as described by Li et al. [46]. Specificity was tested using a standard DFC solution (water), blank plasma and a standard DTE solution (blank plasma). Precision and recovery were tested using 0.25, 5.0, 20.0 μg/mL standard DTE solutions (blank plasma) five times on different days, with five repeats for each concentration. A standard DTE curve was generated using 0.025, 0.05, 0.1, 0.25, 0.5, 1.0, 2.5, 5.0, 10.0, 20.0 μg/mL standard DTE solutions (blank plasma).

### PD of CS in *H. parasuis*

#### MIC, MBC and MPC determination

The CS MIC of *H. parasuis* was determined using a modified broth micro-dilution method, recommended by the Clinical Laboratory Standards Institute (CLSI; Wayne, PA, USA) [47] and Zhang et al. [41]. Approximately 100 μL TSB medium or plasma medium containing CS with degrees of 1.5625, 3.125, 6.25, 12.5, 25, 50, 100, 200, 400, and 800 ng/mL were made up in in 96 well plates. To each treatment, 5 μL diluted *H. parasuis* culture (1.0 × 10^7^ CFU/mL) in the exponential phase was added. A standard *E. faecalis* (ATCC 29212) served as a quality control and was supplemented with ampicillin at 0.5–2 μg/mL. The 96 well plate was agitated at 190 rpm at 37 °C and observed at 24 and 48 h. The concentration of clear well observed by naked eye was considered as MIC.

To determine the MBC, 100 μL medium (TSB medium and plasma medium) from the clear wells, from the MIC test were spread onto TSA plates, and cultured for 48 h at 37 °C. The minimal concentration of bacteria-free (no colonies on the plate) was considered the MBC.

To determine the MPC, TSA plates were prepared by adding CS at a degree increased by a certain amount from MIC. Exponential growth phase bacteria were pelleted by centrifuging at 3000 rpm at 4 °C. The pellet was then diluted to 3 × 10^10^ CFU/mL with TSB medium. From this, 100 μL bacterial solution was applied to TSA plates and incubated for 24 h at 37 °C. After this period, all colonies were counted. The CS provisional MPC (MPCpr) was determined as the lowest concentration after 72 h of sterile growth. The same TSA plates series was prepared and acted with a CS degree decreased by 20% from MPCpr, and MPC was determined as the lowest concentration after 72 h of sterile growth (no colony in plate).

#### In vitro PAE determination

Approximately 1.8 mL exponential phase *H. parasuis* (1.0 × 10^8^ CFU/mL) was mixed with 0.2 mL CS solution, to generate final concentrations of 1 MIC, 2 MIC and 4 MIC. A 0.2 mL aliquot of physiological saline was used as control. Volumes were cultured in glass tubes and grown for 2 h in 37 °C to induce PAE production. 100 μL cultured medium was mixed with 0.9 mL TSB medium and cultured at 37 °C. 100 μL samples were taken at 0, 1, 2, 4, 6, 8, and 12 h, and diluted to 0.1% in sterile physiological saline to count cells. Each treatment was performed four times. Growth curve for *H. parasuis,* at different CS concentrations were established, and T (time required for bacterial numbers to be 10 times higher than 0 h in the test groups) and C (time required for the bacterial numbers to be 10 times higher than 0 h in control groups) values were calculated. PAE was calculated as the difference between T and C (PAE = T - C).

#### In vitro and ex vivo bactericidal curves

To determine in vitro bactericidal curves, exponential growth phase bacteria were diluted to 1.0 × 10^5^ CFU/mL in TSB medium. 1.5 mL of this solution was mixed with 1.5 mL CS solution to generate final concentrations of 1/4, 1/2, 1, 2, 4, 8, 16, and 32-fold MIC in 5 mL glass tubes. 1.5 mL physiological saline was used as 0 MIC. Tubes were incubated at 37 °C for 24 h, with 100 μL removed at 0, 1, 2, 4, 8, 12, and 24 h, and then plated on TSA for colony counting.

To determine ex vivo bactericidal curves, bacteria (1.0 × 10^5^ CFU/mL) were co-incubated with plasma samples from pigs at different CS time points, from piglet sampling. Plasma was prepared by centrifugation (4500 rpm for 5 min), filtered (0.45 μm membrane), inactivated (56 °C for 30 min) and finally filtered (0.22 μm membrane) before being cultured with bacteria at 37 °C for 24 h. 100 μL sample of each treatment was collected at 0, 1, 2, 4, 8, 12, and 24 h, and then applied to TSA plates to count colonies.

### Data processing and the establishment of a PK/PD model

The area under the curve at 24 h; AUC_24 h_/MIC for CS to induce antibacterial and bactericidal effects was determined by fitting a Sigmoid E_max_ model (the Hill equation) with ex vivo PD and in vivo PK parameters, using WinNonlin® software (SCI Software, Statistical Consultants Inc.). This model is described as:

$$ \mathrm{E}={E}_{max}-\frac{\left({E}_{max}-{E}_0\right)\times {C}_e^N}{C_e^N+{EC}_{50}^N} $$

E_max_ represents the maximum difference between the logarithmic value of the number of bacteria before and 24 h after CS administration in the blank group; E_0_ is the maximum difference between the logarithmic value of the number of bacteria in the plasma sample; EC_50_ represents the 50% PK/PD parameter that produced the greatest antibacterial effect in plasma samples; C_e_ is the parameter value of different samples; N represents the slope of the equation and determines the steepness of the S-curve.

### Predicting the dosing regimen

To calculate the dose needed, the AUC_24 h_/MIC of different antibacterial effects was substituted into the following formula:

$$ \mathrm{Dose}\left(\mathrm{perday}\right)=\frac{\left( AUC/ MIC\right)\times MIC\times CL}{F\times {f}_u} $$

where AUC/MIC is the targeted end point for optimal efficacy; MIC is the minimum inhibitory concentration; CL is the clearance per day; f_u_ is the free fraction of drug in plasma (ignore if minimal binding). The ex vivo antibacterial effects of CS administration were quantified at three levels: (1) bacteriostatic action (no change in bacterial counts, E = 0); (2) bactericidal action (99.9% reduction in bacterial counts, E = − 3); and (3) bacterial elimination (99.99% reduction, E = − 4) [39].

### Data processing

PD and some PK data were processed using Excel™ (Microsoft, Redmond, WA, USA). Several PK parameters were also calculated using WinNonlin. The PK data for CS in healthy pigs and *H. parasuis*-infected pigs were analyzed using SPSS v21 (IBM, Armonk, NY, USA).

## Data Availability

The datasets during and/or analysed during the current study available from the corresponding author on reasonable request.

## Abbreviations

CS: Ceftiofur Sodium
DFC: Desfuroylceftiofur
HPLC: High performance liquid chromatography
MBC: Minimum bactericidal concentrations
MIC: Minimum inhibitory concentrations
MPC: Mutant prevention concentrations
PAE: Post-antibiotic effect
PK/PD: Pharmacokinetics/pharmacodynamics
